# Non-Woven Fabrics Based on Nanocomposite Nylon 6/ZnO Obtained by Ultrasound-Assisted Extrusion for Improved Antimicrobial and Adsorption Methylene Blue Dye Properties

**DOI:** 10.3390/polym13111888

**Published:** 2021-06-06

**Authors:** M. Andrade-Guel, C. A. Ávila-Orta, C. Cabello-Alvarado, G. Cadenas-Pliego, S. C. Esparza-González, M. Pérez-Alvarez, Z. V. Quiñones-Jurado

**Affiliations:** 1 Centro de Investigación en Química Aplicada, Saltillo 25294, Mexico; marlene.andrade@ciqa.edu.mx (M.A.-G.); carlos.avila@ciqa.edu.mx (C.A.Á.-O.); pamarissa@hotmail.com (M.P.-A.); 2 CONACYT—Centro de Investigación y de Innovación del Estado de Tlaxcala, Tlaxcala 90000, Mexico; 3 Facultad de Odontología, Unidad Saltillo, Universidad Autónoma de Coahuila, Saltillo 25125, Mexico; sceciliaesparza@gmail.com; 4 Facultad de Ciencias Químicas, Universidad Juárez del Estado de Durango, Durango 34120, Mexico; zoevineth@gmail.com

**Keywords:** non-woven fabric, nylon 6, zinc oxide, antimicrobial

## Abstract

Approximately 200,000 tons of water contaminated with dyes are discharged into effluents annually, which in addition to infectious diseases constitute problems that afflict the population worldwide. This study evaluated the mechanical properties, surface structure, antimicrobial performance, and methylene blue dye-contaminant adsorption using the non-woven fabrics manufactured by melt-blowing. The non-woven fabrics are composed of nylon 6 (Ny 6) and zinc oxide nanoparticles (ZnO NPs). The polymer nanocomposites were previously fabricated using variable frequency ultrasound assisted-melt-extrusion to be used in melt-blowing. Energy dispersion spectroscopy (SEM-EDS) images showed a homogeneous dispersion of the ZnO nanoparticles in nylon 6. The mechanical properties of the composites increased by adding ZnO compared to the nylon 6 matrix, and sample Ny/ZnO 0.5 showed the best mechanical performance. All fabric samples exhibited antimicrobial activity against *S. aureus* and fungus *C. albicans*, and the incorporation of ZnO nanoparticles significantly improved this property compared to pure nylon 6. The absorption efficiency of methylene blue (MB), during 60 min, for the samples Ny/ZnO 0.05 and Ny/ZnO 0.25 wt%, were 93% and 65%, respectively. The adsorption equilibrium data obeyed the Langmuir isotherm.

## 1. Introduction

There has been a continued increase in environmental problems due to the contamination of water by at least 10,000 different dyes and pigments that are used in the textile industry [1]. Methylene blue is a cationic dye used for dyeing cotton, wool, and silk. This dye can cause impacts when reaching water resources due to the reduction in sunlight infiltration, in addition to health problems if ingested [2]. The spread of infectious diseases generated by pathogenic microorganisms in water has become one of the most severe public health problems worldwide. This issue has triggered social, scientific, and industrial interest in the development of practical alternatives that contribute to the decrease in harmful agents. Recently, the design and application of polymers with ceramics nanoparticles has generated valuable results in areas related to health and personal care, since the incorporation of nanometric charges drastically modifies the antibacterial properties of the materials obtained [3,4].

Different materials have been reported for the adsorption of dyes between them magnetite nanoparticles [5], urea calcium alginate xerogel beads [6], metal/mineral materials such as CuO, zinc, iron, and zeolite [7]. Bioadsorbents have also been used as bivalve shells of anadara uropigimelana [8], sepia shells (cuttlefish bones) [9], to fabricate monolithic algal green powder (MAGP) based on the marine green macroalga enteromorpha flexuosa [10] as well as magnetically modify products of the coffee industry [11]. Nowadays, methodologies based on energy sources such as microwave or ultrasound radiation are exploited to accelerate different sorption processes. These technologies are considered green, efficient and more time efficient. Most of the adsorbent materials mentioned above need to be regenerated, which does not solve the problem of water contamination since organic solvents are needed for the regeneration of said material and only transfer the problem of contaminants to the eluent in this case, the solvent used for the regeneration [12].

Filtration methods are promising technologies to remove contaminants in water [13]; an alternative is the use of non-woven fabric obtained from a polymer nanocomposite. Nylon 6 is a common raw material for textile fiber or fabrics that can be elastic, resistant, and prevents moth attack. These fibers are used in the manufacture of socks, fabrics, knitted fabrics, and bristles [14]. Moreover, cotton fabrics coated with ZnO nanoparticles showed satisfactory results against Gram-positive *Staphylococcus aureus* and Gram-negative *Escherichia coli*. However, these fabric coatings are on the surface of the fibers so they can be lost over time or after several washing cycles [15].

Constant washing affects both the antimicrobial properties and the toxicity of the antimicrobial agents in commercial antimicrobial fabrics. ZnO is a ceramic with antimicrobial, antifungal properties, environmental stability, and its use is approved by the Food and Drug Administration (FDA) [16].

The exact mechanism of antibacterial activity remains controversial, since there are still doubts as to how ZnO nanoparticles act [17]. Some of the mechanisms that have been proposed in the literature are: direct contact of ZnO nanoparticles with cell walls, which results in the destruction of bacterial cells and the disruption of DNA replication [18], the release of antimicrobial ions, mainly Zn^2+^ [19], and ROS formation [20], whilst other authors they attribute it to the effects of different conditions on the antibacterial activity such as the size of the nanomaterials, concentration/dosage, temperature, capping agent, and reducing agent [21]. Most studies have shown the strong ROS generating potential of ZnO nanoparticles, suggesting it has an important role in bacterial killing.

On the other hand, one of the methods for the production of non-woven fabric is melt-blowing, which is a technique with several advantages compared to the electrospinning method, such as it is scalable, it does not require solvents, and it is a single-process step that produces fiber diameters from 0.3 to 20 µm [22].

The originality of the study is based on the fact that the ultrasound-assisted extrusion process homogenizes the distribution of the nanometric particles in the polymer matrix, requesting to have a non-woven fabric with isotropic properties. In other studies, the incorporation of ZnO in textiles is carried out by functionalization with a textile substrate by dip coating methods, sol–gel and electrodeposition, among others [23]. This work reports the manufacture of non-woven nylon 6 fabric with ZnO nanoparticles forming nanocomposites prepared by an ultrasound-assisted extrusion process, using different concentrations of nanometric ZnO particles (0.25, 0.5, and 1.0 wt%). Morphology, the mechanical properties, antibacterial activity, and methylene blue adsorption of these non-woven fabrics were evaluated leading to the proposal of not only the adsorption of the dye but also its degradation.

## 2. Materials and Methods

Zinc oxide nanoparticles were purchased from SkySpring Nanomaterials Inc. (Houston, TX, USA), with an average diameter of 15–30 nm, hemispherical geometry, and 99.98% purity. A commercial nylon 6 resin from DuPont Zytel 7301 NC010 (Wilmington, DE, USA) was used as the polymer matrix. Methyl blue (MB) was purchased from Sigma Aldrich (Saint Louis, MO, USA), classification Acute Tox. 4; H302.

### 2.1. Synthesis of Nylon 6/ZnO Nanocomposites

Nylon 6/ZnO nanocomposite preparation was carried out in the following way: nylon 6 polymer with three charges of ZnO nanoparticles (0.25%, 0.5%, and 1%) were prepared by an ultrasound-assisted melt extrusion process. A laboratory-size twin-screw extruder from Thermo Scientific (model, Prism TSE-24MC) (Thermo Fisher Scientific, Waltham, MA, USA) with a screw diameter of 24 mm and L/D ratio of 40:1 was assisted by a catenoidal ultrasonic tip (Branson Ultrasonics Corp., Brookfield, CT, USA) [24]. The temperature profile was 235 °C and the rotational speed was 100 rpm. The extruder was connected to a homemade ultrasonic generator (15 to 50 kHz, 100% of 750 W). Previously, the resin was dried for 12 h at 100 °C before the preparation of nanocomposites.

### 2.2. Synthesis of Non-Woven Fabrics

The non-woven fabric was manufactured using a Fiber Extrusion Technology (FET-UK) pilot machine, equipped with two single spindle extruders. The process conditions of non-woven fabric made from nanocomposites nylon 6/ZnO, were: extrusion zone 1, 245 °C, extrusion zone 2, 250 °C, extrusion zone 3, 270 °C, extrusion zone 4, 270 °C, flange zone, 275 °C, melt pump zone 275 °C, dual heat zone 275 °C, melt-blown adapter zone, 280 °C, melt-blown hot air zone, 280 °C. The speed of the collecting band was 0.6 m/min, and the speed of the dosing pump was 6 and 8 rpm. The use of two different extruders allows the preparation of the nanocomposite separately to subject it to an ultrasound-assisted extrusion that disperses the nanoparticles in the polymeric matrix. Once that the nanocomposite is obtained; the non-woven fabric is manufactured in the FET-UK extruder by melt-blow.

**Figure 1:**
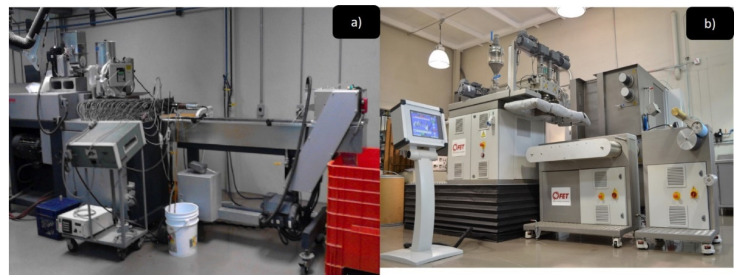
Images of extruders used in to manufacture nonwoven fabric: (**a**) twin-screw extruder connected to a homemade ultrasonic generator; and (**b**) fiber extrusion technology (FET-UK) pilot machine.

Samples of pure nylon 6 non-woven fabric and nylon 6/ZnO nanoparticles non-woven fabric at 0, 0.25, 0.5 and 1.0 wt%, were identified as: Ny; Ny/ZnO 0.25; Ny/ZnO 0.5; and Ny/ZnO 1, respectively. Figure 1 shows the two types of extruders used to manufacture these materials: first the nanocomposites; and later the non-woven fabrics.

### 2.3. Characterization

The non-woven nylon 6/ZnO fabrics were observed using a Carl Zeiss HD HAL/LED optical microscope. The surface analysis was done by scanning electron microscopy and energy dispersion spectroscopy (SEM-EDS) through an electron microscope model JEOL JSM-7401F; the microscope acceleration voltage was 3.0 kV using the LEI secondary electrons as a detector. The mechanical tests were carried out by the universal machine CEF-122, according to ASTM-D-5034-09 and ASTM-D-2261-13.

Accelerated washing tests were carried out under the AATCC-TEST METHOD 61 standard for all the non-woven fabrics obtained.

The antimicrobial activity of the materials was evaluated through the agar diffusion technique based on Kirby Bauer. This method determines the sensitivity of a microorganism against a specific material: in this case, non-woven fabric [25].

The antimicrobial tests were carried out as follows: strains *Candida albicans* (ATCC^®^ 10231™), and *Staphylococcus aureus* (ATCC^®^ 23235™) were grown in a liquid medium—nutritious broth for *C. albicans* and soy trypticase broth for *S. aureus*. The bacteria were incubated at 37 °C and the fungus, at 33 °C for 24 h, until turbidity was obtained in the middle of 0.5 Mc Farland each of the inoculums. Grown cultures were prepared and smeared onto the surface of nutrient agar and soy trypticase in Petri plates. Specimens of approximately 5 × 3 mm in size were cut from the textile samples under aseptic conditions and placed in the agar surface and the plates were incubated at 37 °C and 33 °C, respectively, for 24 h. and the diameter of the formed zone of inhibition (in mm) was determined.

For evaluating the adsorption of dye, the fabrics were activated with a UV lamp (Numak model GL-9406) at a wavelength of 365 nm irradiating for 10 min. The adsorption experiments were carried out by filtering 20 mL of the 200 mg/L solution (methylene blue) with non-woven fabric (5 × 5 cm) with a weight of 20 mg at room temperature during 60 min. The non-woven fabric was fixed in a glass funnel and the methylene blue solution was poured into the non-woven fabric slowly for 60 min.

An aliquot (solution methylene blue) of the experiment was taken every 15 min to be analyzed by UV–Vis spectroscopy at 664 nm. All the experiments were made in triplicate.

The percentage of adsorption efficiency was calculated according to Equation (1):

(1) $$\%\;Adsorption\;efficienty=\frac{C_{i}-C_{e}}{C_{i}}\times 100$$

where *C_i_* and *C_e_* are the initial and final concentrations, respectively.

The adsorption capacity of the non-woven fabric was calculated with Equation (2) in equilibrium:

(2) $$q_{e}=\frac{(C_{i}-C_{e})V}{m}$$

where *V* is the volume of the solution in liters (L) and *m* is the amount of mass in mg of absorbent.

#### Adsorption Isotherm

The Langmuir and Freundlich models were used to describe the adsorption equilibrium data. For the assessment of both models, the absorption isotherms’ data were fitted and the correlation coefficient (*R*^2^) was calculated using the trendline command in Microsoft Excel.

The Langmuir isotherm was calculated by Equation (3):

(3) $$\frac{C_{e}}{q_{e}}=\frac{C_{e}}{q_{m}}+\frac{1}{K_{L}q_{m}}$$

where *q_e_* (mg·g^−1^) and *C_e_* (mg·L^−1^) are the concentrations of the solid and liquid phases of adsorbate in equilibrium, respectively, *q_m_* is the maximum adsorption capacity, and *K_L_* is the constant obtained from the graph of *C_e_*/*q_e_* against *C_e_*.

The Freundlich isotherm was calculated by Equation (4):

(4) $$\ln q_{e}=\ln K_{F}+\left(\frac{1}{n}\right)\ln\;C_{e}$$

where *K_F_* (mg·g^−1^) (L·mg^−1^) and 1/*n* are the Freundlich constants related to the adsorption capacity and *n* is the heterogeneity factor calculated by linearly plotting ln·*q_e_* against ln·*C_e_*.

### 2.4. Nonwoven Fabric Regeneration

After the adsorption of methylene blue, the non-woven fabric turns blue. To regenerate the non-woven fabric and reuse it, it was exposed to the sunlight for 3 h or under a UV lamp (Model Numak GL-9406) with a wavelength of 365 nm for one hour. After treatment, the non-woven fabric removed the blue color, which indicates the degradation of the MB.

## 3. Results and Discussion

### 3.1. Microscopy Characterization of Nylon 6 and Nanocomposites

**Figure 2:**
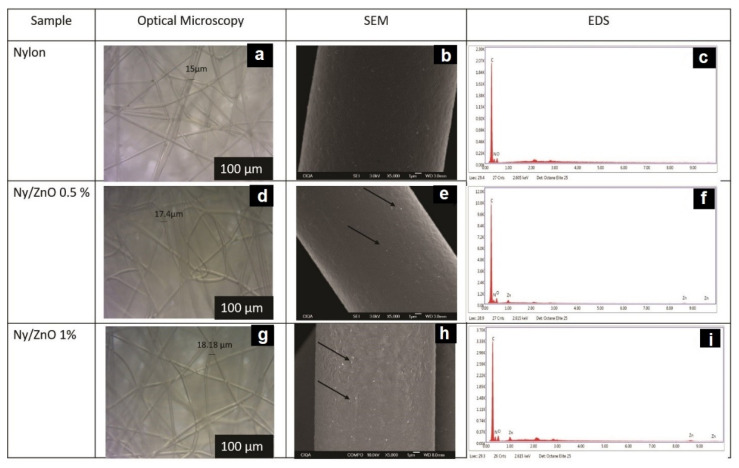
Optical microscopy of (**a**) nylon; (**d**) Ny/ZnO 0.5; and (**g**) Ny/ZnO 1. SEM images of (**b**) nylon; (**e**) Ny/ZnO 0.5; and (**h**) Ny/ZnO 1. EDS spectra of (**c**) nylon; (**f**) Ny/ZnO 0.5; and (**i**) Ny/ZnO 1.

Optical microscopy images of nylon 6 and the nanocomposites (Figure 2a,d,g) show that the fibers maintain a random orientation and present a cylindrical shape with a diameter of 15–18.1 microns, though no significant differences were observed between the samples. SEM micrographs show that the fibers did not exhibit damage on their surface, observing only some roughness (Figure 2b,e,h). Nanocomposite micrographs show a ZnO NPs homogeneous dispersion on the fiber surface, which is according to the concentration employed. EDS spectra (Figure 2f,i) confirm the presence of ZnO NPs. The evidence observed by the SEM technique suggests that NPs can play an important role in the degradation of MB because their surface can interact with the dye and cause its degradation. Raza et al. [26] incorporated ZnO with chitosan into cotton fabric, finding a prevalence of the zinc element, which is in agreement with this study they also observed the agglomeration of ZnO particles due to the impregnation method used.

### 3.2. Mechanical Properties

**Figure 3:**
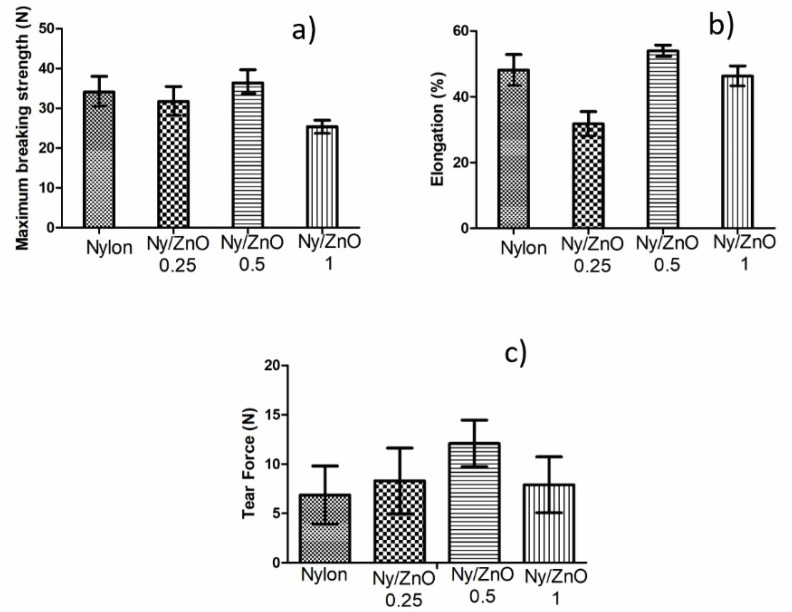
Mechanical properties of non-woven fabrics: (**a**) maximum breaking strength; (**b**) elongation %; and (**c**) tear force.

Figure 3 shows the results of the mechanical analysis of maximum breaking strength, elongation percentage, and tear force. Nylon 6 presented a maximum breaking strength of 31.87 MPa, which increased to 35.67 MPa for the sample with 0.5%wt. of ZnO. This behavior is related to previous studies, which refers that a low amount of ZnO and an adequate dispersion of the nanoparticles improved the mechanical properties of nanocomposites from polyamide and polyurethane [27]. While the behavior for elongation and tear force was similar to the previous measurement, the sample Ny/ZnO 0.5 showed the best performance.

### 3.3. Antimicrobial Properties

**Figure 4:**
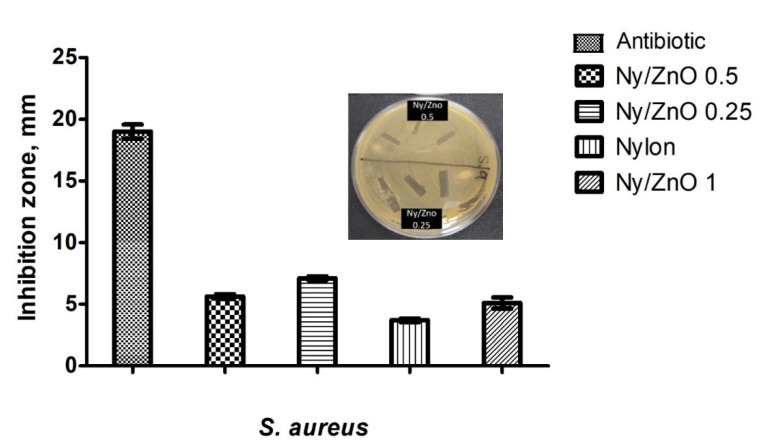
Effect of control samples, antibiotic, nylon, Ny/ZnO 1, Ny/ZnO 0.5, Ny/ZnO 0.25 on the growth of *S. aureus*.

**Figure 5:**
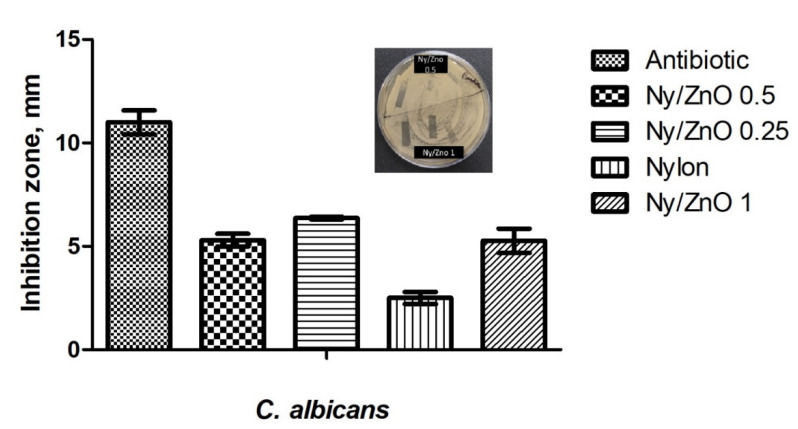
Effect of antibiotic, nylon, Ny/ZnO 1, Ny/ZnO 0.5, Ny/ZnO 0.25 on the growth of *C. albicans*.

The antimicrobial activity of non-woven fabrics was tested using the Kirby Bauer agar diffusion method. The macroscopic results reveal a contact inhibition effect using Ny/ZnO materials. Figure 4 shows a complete inhibition for *S. aureus*; however, nylon without nanoparticles also presents a growth inhibition, which has already been reported by Mowery et al. [28]. These authors attribute the biological activity of nylon to the fact that the polymer has a structural basis of the oligomers of β-peptides made up of amino acids, which damage the cell wall. Any sample of non-woven fabric shows inhibition against *E. coli*, however, the samples of Ny/ZnO 0.25, Ny/ZnO 0.5, and Ny/ZnO 1.0 of non-woven fabric show inhibition against the fungus *C. albicans* (Figure 5). Janaki et al. [29] report activity against *C. albicans* of ZnO using the diffusion method in agar; they attribute the inhibition to the formation of reactive oxygen species (ROS) such as superoxide anion O_2_^−^, hydrogen peroxide H_2_O_2_ and hydroxyl radical (HO**^·^**), generated by the ZnO. These reactive species damage the cells of the microorganisms resulting in their decomposition and entire destruction and the Zn^+2^ ions have a minor influence on the antibacterial activity [30].

Dural Erem et al., when obtaining fibers with nylon 6 and ZnO nanocomposites in a 16 cm long microcomponder, feeding 10 g of the nanocomposite. They found that the sensitivity barrier in its fibers can vary depending on the ZnO content and also by the type of bacteria. In addition, they observed that the bacteriostatic effect of the fibers against Gram-positive bacteria was presented at 0.5 wt% ZnO particles and this same effect against Gram-negative bacteria was obtained at a concentration lower than 0.5 wt%. These authors tested concentrations up to 5 wt% [31].

### 3.4. Methylene Blue Adsorption

**Figure 6:**
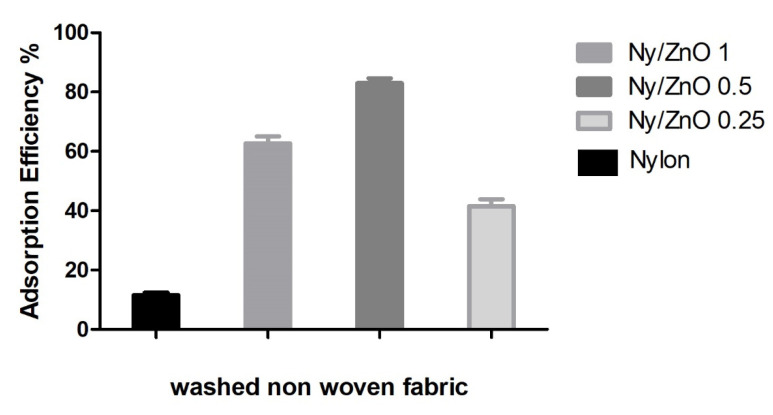
The adsorption efficiency of nylon, Ny/ZnO 1, Ny/ZnO 0.5, Ny/ZnO 0.25 washed non-woven fabric (MB concentration = 200 mg/L; nanocomposite content = 20 mg/20 mL; T = 25 °C and t = 60 min).

After an accelerated wash treatment using the AATCC-TEST METHOD 61 standard to evaluate the color fastness and changes in the surface of the fabric, using detergents and washes, the fabrics were characterized to determine the adsorption efficiency of methylene blue, when using nylon 6 non-woven fabric with ZnO nanoparticles. Figure 6 shows the adsorption efficiency percentage, where we can observe that the pristine nylon non-woven fabric only presented 13% adsorption efficiency, indicating that methylene blue could not be removed from the water by the non-woven fabric without nanoparticles. This agrees with that reported by Lee et al., in the adsorption of methylene blue using nylon-6 nanofibers entangled with graphene flakes. The nylon 6 presents low adsorption, and by increasing the concentration of graphene, it presents a higher performance [32].

Non-woven fabrics assigned as Ny/ZnO 0.25, Ny/ZnO 0.5 and Ny/ZnO 1.0 presented percentages of adsorption of methylene blue of 42%, 82% and 61%, respectively. Ummartyotin et al. reported the synthesis of ZnO nanoparticles which were deposited on the surface of nylon 6 by the spin-coated method: they reported only 60% adsorption of methylene blue using different percentages of the weight of nanoparticles 1.0, 3.0 and 5.0 wt% [33]. In this study, we report 82% adsorption of methylene blue for the Ny/ZnO 0.5 sample which contains 0.5% of ZnO nanoparticles. This is attributed to the nanocomposite preparation by means of ultrasound-assisted extrusion that allows a uniform distribution and a good dispersion of the nanoparticles in non-woven fabric. On the other hand, with the other studies, they used impregnation methods, dip-coating, spin-coating that creates agglomerates on the surface and with washing the coating that was acquired can be eliminated. A different situation occurs in the non-woven fabrics prepared in this study: the ZnO NPs are inside the polymer matrix and are not removed.

In another study, the color removal of textile wastewater was achieved up to 81%, with only ZnO nanoparticles synthesized using a polyvinylpyrrolidone (PVP)-assisted co-precipitation route [34]. This method can be limited because the nanoparticles are not supported in a polymeric matrix and have to directly come into contact with the wastewater from the textile industry. The method proposed in this paper is considered a method that can be very useful at an industrial level, since it uses low amounts of ZnO nanoparticles and can be adaptable to large volumes of wastewater. The advantage of using non-woven fabric for dye removal in water is that it is used as a reusable filter and can also retain solids and micrometer-sized particles.

#### 3.4.1. Effect of UV Radiation on MB Adsorption

**Figure 7:**
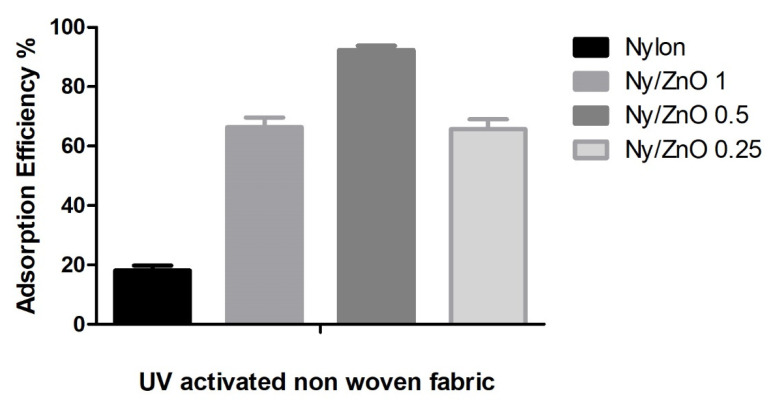
The adsorption efficiency of nylon, Ny/ZnO 1, Ny/ZnO 0.5, Ny/ZnO 0.25 UV activated non-woven fabric (MB concentration = 200 mg/L, nanocomposite content = 20 mg/20 mL, T = 25 °C and t = 60 min).

Figure 7 shows the adsorption efficiency when the non-woven fabric is activated for 10 min with a UV lamp, an increase in the percentage of adsorption efficiency is observed in all samples. Ny/ZnO 0.5 presents 93% adsorption efficiency, and an increase in the percentage of adsorption efficiency is also observed in the Ny/ZnO 0.25% sample from 40% to 65%. Although the exposure time to UV light is short, the non-woven fabric that contains the ZnO nanoparticles can be activated even though the ZnO concentrations are low and these are dispersed in the polymeric matrix. Another work reported by Ashraf et al. details the use of long exposure times to UV radiation (4 h), for the polyester fabric functionalized with the ZnO nanorods system. In the Ny/ZnO 1 sample, there is only an increase of 4% regarding adsorption efficiency when the sample is UV irradiated, which may be due to the concentration of ZnO and a blockage caused by more molecules adsorbing active sites [35].

#### 3.4.2. Adsorption Efficiency of MB as a Function of Time

**Figure 8:**
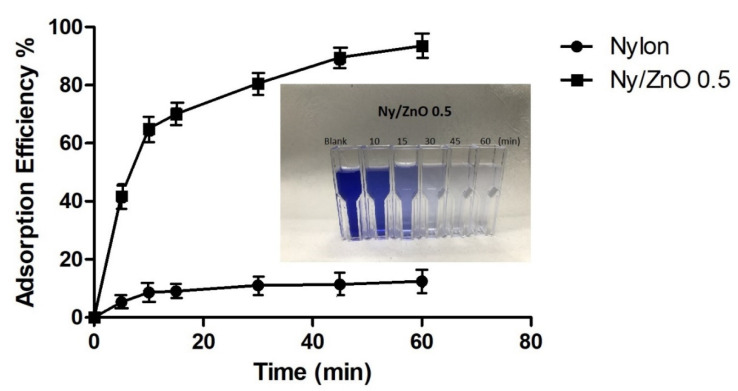
The adsorption efficiency of nylon and Ny/ZnO 0.5 (MB concentration = 200 mg/L, nanocomposite content = 20 mg/20 mL, T = 25 °C and t = 60 min).

The adsorption efficiency of methylene blue according to the time of the nylon and Ny/ZnO 0.5 samples is shown in Figure 8. The non-woven nylon fabric without nanoparticles showed low levels of adsorption, and after 60 min, the maximum adsorption was 18%. For non-woven fabric with 0.5 wt% of ZnO nanoparticles, there is a higher adsorption efficiency (95%). The great adsorption efficiency of the non-woven Ny/ZnO fabric can be attributed to a good dispersion of the NPs in the polymer matrix, and the synthesis of nanocomposites of nylon 6 using an ultrasound-assisted extrusion process is an effective method of dispersing different nanoparticles [36,37], which is also conserved in the non-woven fabric. The adsorption properties are preserved even after washing, and the discoloration can be observed in the image of Figure 8 with the exposure time of the Ny/ZnO 0.5 sample.

#### 3.4.3. Adsorption Isotherm

**Figure 9:**
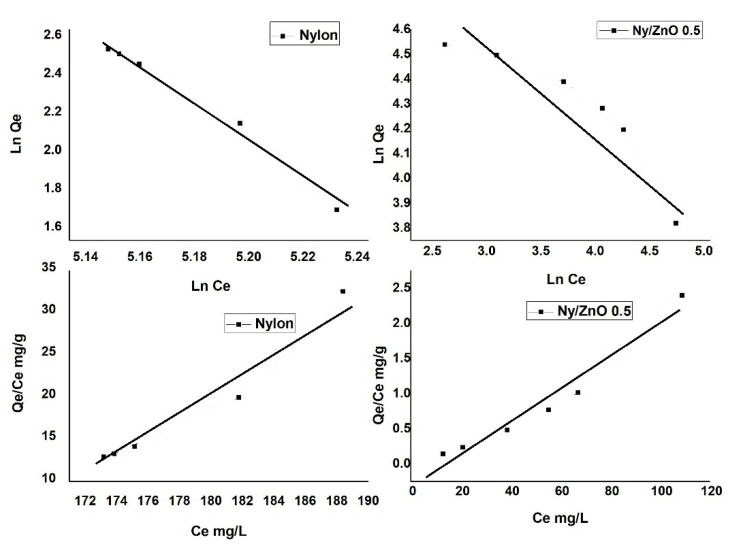
Langmuir and Freundlich model of adsorption for methylene blue of the nylon and Ny/ZnO 0.5 (MB concentration = 200 mg/L, nanocomposite content = 20 mg/20 mL, T = 25 °C and t = 60 min).

The results of the Langmuir and Freundlich isotherms are presented in Figure 9. The Ny/ZnO 0.5 isotherm has a homogeneous adsorption behavior, when presenting the coefficient of determination (*R*^2^) value of 0.95 (Table 1), which agrees with that reported by Jawad et al. [38]. The Langmuir isotherm is based on the assumption that an activation point on the surface of the adsorbent is capable of adsorbing a molecule, indicating the formation of a monolayer [39,40]. On the other hand, the nylon sample has a heterogeneous adsorption behavior when presenting the coefficient of determination (*R*^2^) value of 0.98. The Freundlich isotherm is based on a multilayer adsorption, and the concentration on the surface of the adsorbent increases as the adsorbate concentration increases [36].

#### 3.4.4. Effect of pH on the Adsorption MB

**Figure 10:**
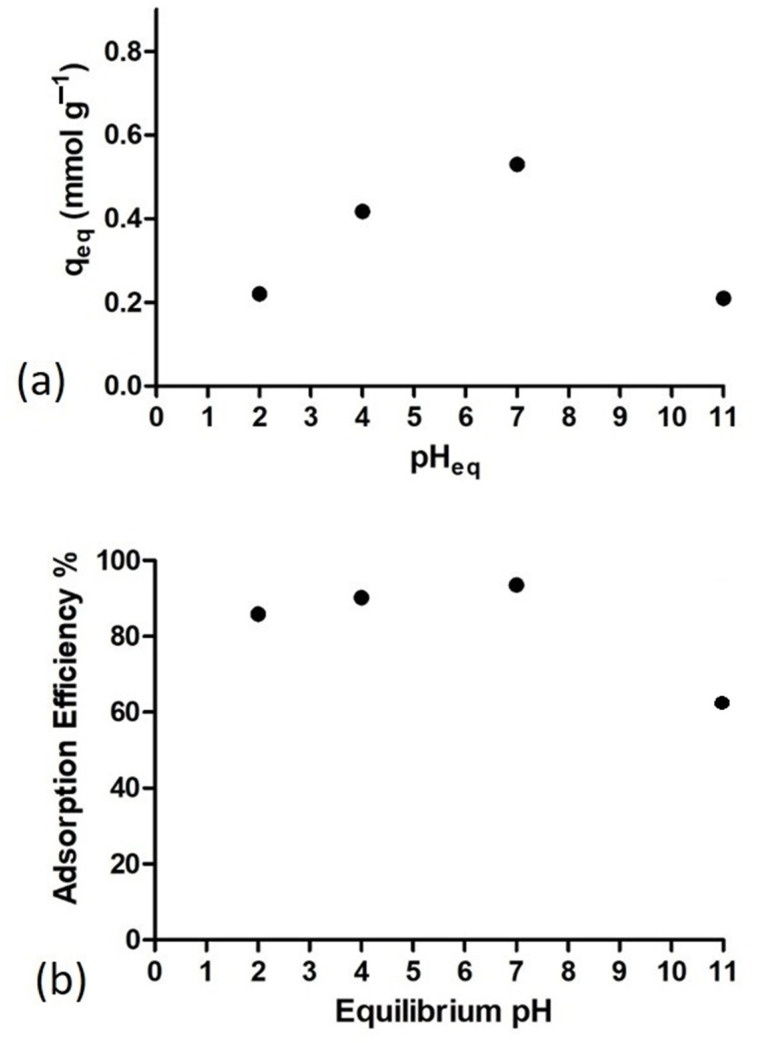
(**a**) Effect of pH on methylene blue of adsorption onto non-woven fabric Ny/ZnO 0.5; (**b**) adsorption efficiency % a different pH (MB concentration = 200 mg/L, nanocomposite content = 20 mg/20 mL, T = 25 °C and t = 60 min).

The pH is a parameter that can affect the adsorption capacity of the material since it plays an important role in protonation/deprotonation, though it can also affect the affinity of the adsorbent surface. Figure 10a shows the effect of pH on the adsorption capacity of the Ny/ZnO non-woven fabric for methylene blue: from pH 2, to 4 and 6, the adsorption increases progressively; at pH 11, the adsorption of the dye is reduced. In Figure 10b, the adsorption efficiency increases from 80% to 93% when the pH increases from 2 to 7, and in the case of a basic pH such as 11, there is a decrease of 60%. Elwakeel et al. reported sorption efficiency, >92% of MB for chitosan functionalized with 2-mercaptobenzimidazole, they present a linear behavior [41]. Instead of another study for the removal of methylene blue using bivalve shells of anadara uropigimelana as a biosorbent at pH 11 and 12, there is a decrease in the percentage of the removal or adsorption similar to that reported in this work [8].

#### 3.4.5. Mechanism of the Adsorption

**Figure 11:**
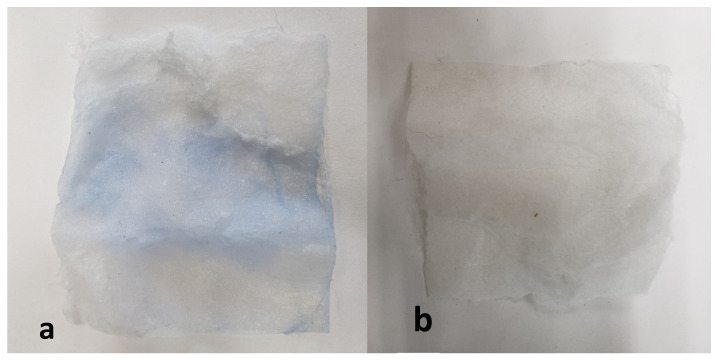
(**a**) Non-woven fabric with MB; and (**b**) non-woven fabric after one hour irradiation with UV lamp.

First, a dye adsorption occurs on the non-woven fabric as seen in Figure 11a; the non-woven fabric turns blue due to the contaminant (dye). In this case, the non-woven fabric is transferred, then it is irradiated by sunlight for 3 h or using the UV lamp for an hour and as shown in Figure 11b, the colorant degrades and is totally eliminated. Unlike the other materials previously reported with the Ny/ZnO nonwoven fabric, no solvent is required for regeneration.

**Figure 12:**
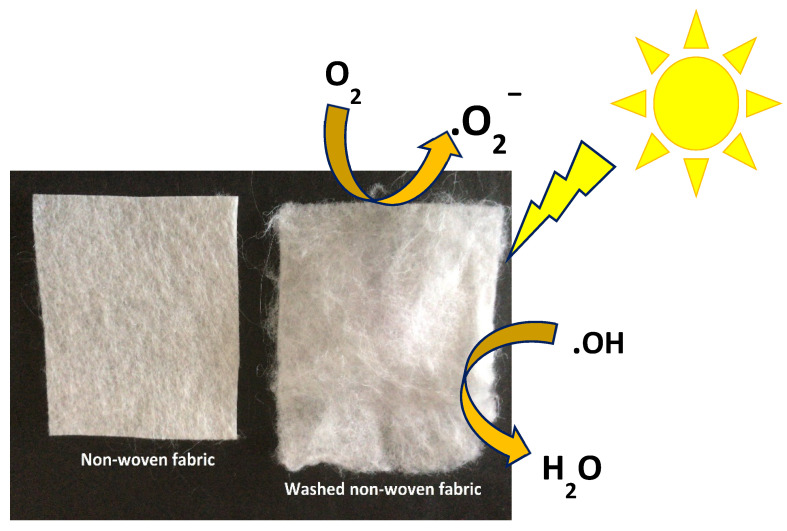
Schematic representations of the degradation mechanism of methylene blue dye on non-woven fabric under UV irradiation.

Figure 12 shows the methylene blue degradation scheme. The non-woven fabric is irradiated with a UV lamp, which generates free radicals ˙OH and ˙O_2_ due to the presence of the ZnO nanoparticles in the polymeric matrix. The photocatalytic activity of ZnO increases when irradiating for 10 min with the lamp. These two types of radicals decompose methylene blue [42], and in addition to that, they can break the cell membrane of bacteria, prevent their growth and inhibit them from water.

Unlike other materials, this non-woven fabric can be produced at an industrial level as a water filter, since it can eliminate the microorganisms and dyes present in contaminated water.

## 4. Conclusions

Non-woven fabrics from nylon 6 and ZnO were successfully manufactured by melt-blowing. SEM images showed that the ZnO nanoparticles were dispersed homogeneously into the polymer. The sample Ny/ZnO 0.5 presented the best mechanical performance. All samples inhibited the growth of *S. aureus*, including the nylon matrix. The non-woven fabric prepared with just 0.25 wt% ZnO prevented the growth of *C. albicans*. The methylene blue absorption efficiency was 93% in a time of 60 min., and the adsorption equilibrium data obeyed the Langmuir isotherm.

## Figures and Tables

**Table 1:** Parameters of the isotherm constants and correlation coefficients calculated for urea adsorption.

Sample	Langmuir			Freundlich		
	k	*q*_max_	*R* ^2^	*n*	*K_F_*	*R* ^2^
Nylon	1.20	196.92	0.9387	9.61	52.17	0.9817
Ny/ZnO 0.5	0.023	0.33	0.9527	0.30	5.41	0.8346

## Data Availability

The data presented in this study are available on request from the corresponding author.
